# Grandmothers Raising Grandchildren: Predictors of Healthcare Access and Financial Security During COVID-19

**DOI:** 10.1093/geroni/igab046.1006

**Published:** 2021-12-17

**Authors:** Alexandra Jeanblanc, Chris Burant, Carol Musil

**Affiliations:** 1 Case Western Reserve University, Cleveland, Ohio, United States; 2 CWRU School of Nursing, Cleveland, Ohio, United States

## Abstract

Grandmothers living with or raising grandchildren who had just completed the final data point of an NIH-funded, national, behavioral RCT were asked to complete an additional data collection point to capture the effects of the Covid-19 pandemic on their families’ access to healthcare and financial security. In Spring 2020, 258 grandmothers completed measures of access to healthcare and financial security (3 and 4 item composite scales), family strain, family functioning, and psychosocial and demographic variables. Financial security (Adj. R2=.52) was explained by knowing other grandfamilies; better family functioning; and fewer financial worries, unmet service needs, and depressive symptoms. Access to healthcare (Adj. R2=.24) was explained by being married, employed and having fewer financial worries and unmet service needs. Findings that family functioning, knowing other grandfamilies and depressive symptoms contributed to financial security, and that marital and employment status affect access to healthcare show the importance of support.

